# Case of intestinal obstruction due to accidental ingestion of a silicone rubber dental impression

**DOI:** 10.1093/jscr/rjaf195

**Published:** 2025-05-16

**Authors:** Yuki Takano, Shingo Tachibana, Kazuhiko Kasuya, Keigo Fugami, Yuichi Nagakawa

**Affiliations:** Department of Surgery, Toda Chuo General Hospital, 1-19-3 Honcho, Toda 335-0023, Japan; Department of Gastrointestinal and Pediatric Surgery, Tokyo Medical University, 6-7-1 Nishishinjuku, Shinjuku 160-0023, Japan; Department of Surgery, Toda Chuo General Hospital, 1-19-3 Honcho, Toda 335-0023, Japan; Department of Gastrointestinal and Pediatric Surgery, Tokyo Medical University, 6-7-1 Nishishinjuku, Shinjuku 160-0023, Japan; Department of Surgery, Toda Chuo General Hospital, 1-19-3 Honcho, Toda 335-0023, Japan; Department of Gastrointestinal and Pediatric Surgery, Tokyo Medical University, 6-7-1 Nishishinjuku, Shinjuku 160-0023, Japan; Department of Chemistry, Tokyo Medical University, 6-1-1, Shinjuku, Shinjuku 160-8402, Japan; Department of Gastrointestinal and Pediatric Surgery, Tokyo Medical University, 6-7-1 Nishishinjuku, Shinjuku 160-0023, Japan

**Keywords:** accidental ingestion, foreign body, dental impressions, silicone material, intestinal obstruction

## Abstract

Accidental ingestion is common and can even necessitate surgery. An 89-year-old man with dementia presented with abdominal distension and pain. A CT scan revealed a high-intensity object in the terminal ileum with distension of the proximal small intestine. Colonoscopy showed ileus caused by a retained foreign body (FB), which was removed via laparotomy the following day. Chemical analysis identified the material of the FB as platinum silicone rubber. Based on its material and irregular shape, the FB was determined to be a dental impression. The patient had dentures made one year prior, and cognitive decline may have contributed to the incident. Platinum silicone, which is odorless and does not deform with body heat or digestive juices, maybe a common cause of accidental ingestion.

## Introduction

Foreign body (FB) ingestion is common in daily life, particularly among the elderly, including those with dementia [1, 2]. Recently, a new type of FB has been identified in this group, which had not been previously reported. This case presents an FB that was unidentifiable by the naked eye but was identified through chemical analysis.

## Case report

**Figure 1:**
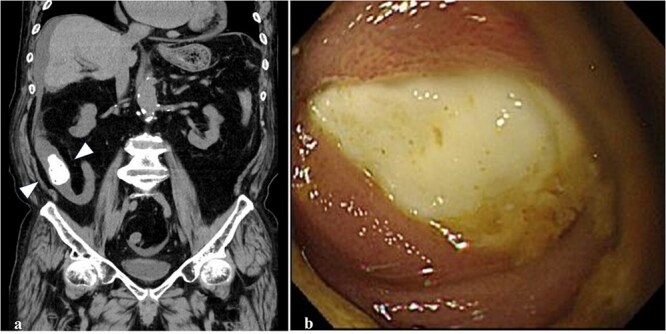
Abdominal CT and colonoscopy. (a) A highly dense mass with fine, low-density spots inside the lumen of the terminal ileum (arrowhead) on the colonal slice of plane CT. (b) The ileum lumen 20 cm proximal from the ileocecal valve was completely occupied by a whitish FB with a hard elasticity and smooth surface when visualized using a colonoscope.

An 89-year-old man with age-related dementia presented with abdominal distension, vomiting, and pain. Physical examination revealed mild abdominal distension and tenderness, especially in the right lower abdomen. Laboratory tests showed an elevated white blood cell count (8430/μl) and C-reactive protein (6.39 mg/dl). Abdominal CT revealed a 30-mm high-density mass in the terminal ileum, with dilated proximal intestines but no signs of intestinal ischemia or free air (Fig. 1a). Based on these findings, the patient was diagnosed with intestinal obstruction due to an FB. Colonoscopy showed a hard, whitish FB 20 cm proximal to the ileocecal valve, which could not be retrieved with a snare (Fig. 1b). Surgery was planned for the second day of hospitalization.

**Figure 2:**
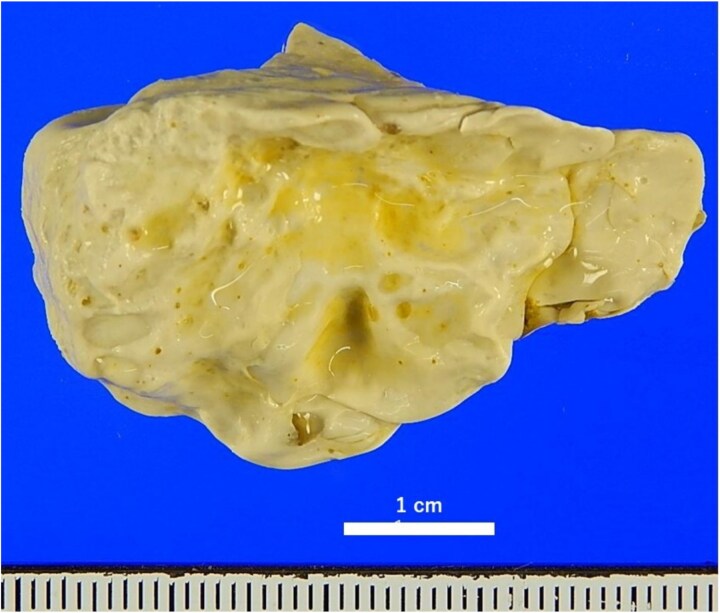
Macroscopic findings of the removed FB after formalin fixation the surface was yellowish-white and irregularly uneven, and the elasticity was similar to that of a plastic eraser.

*Surgical Procedure*: An open surgery was performed after the laparoscopic approach failed due to poor visibility. A 5-cm-diameter mass was palpable 30 cm from the ileocecal valve. Upon incision, a 45 × 30 × 18 mm specimen was removed. The specimen had a smooth, pale-yellow surface and a texture similar to a plastic eraser, suggesting it was a solidified caulking agent (Fig. 2).

**Figure 3:**
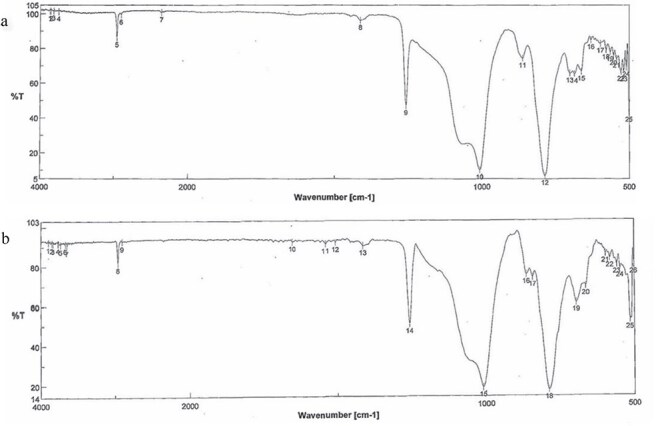
Infrared absorption spectrum. (a) Commercially available silicone rubber. (b) Extracted FB. The patterns of the spectrum wavelengths of both were almost the same. The vertical and horizontal axes show the transmittance (%) and wave number (cm^−1^), respectively. Wave number = reciprocal of wavelength (cm).

**Figure 4:**
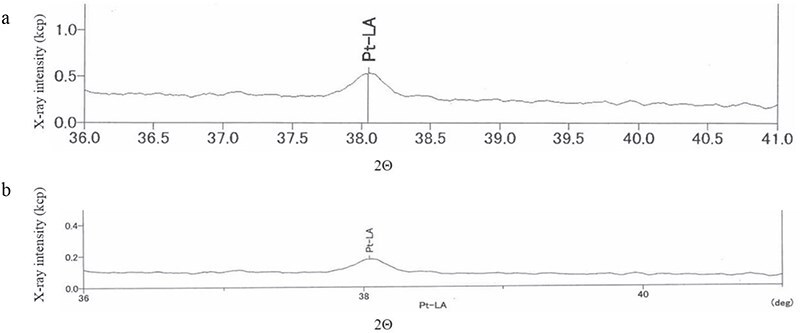
Elemental analysis using X-ray fluorescence diffraction. (a) Platinum (Pt) as a control. (b) Extracted FB. The peak position (2Θ) of the control and FB was 39.045°a) and 38.047°, respectively, and the peak intensity was 0.262 of the X-ray intensity (kcp). The vertical and horizontal axes show kcps and spectral angles of 2Θ, respectively.

**Figure 5:**
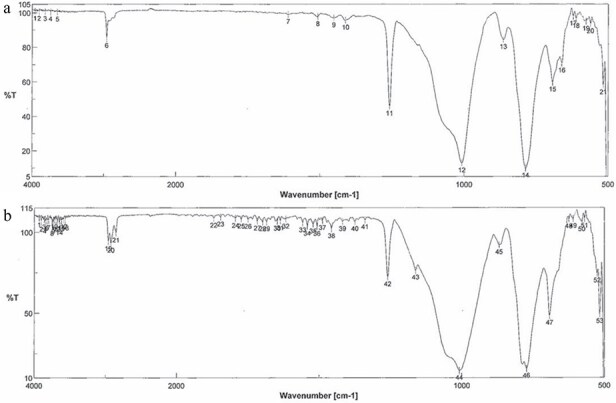
Infrared absorption spectrum. (a) Commercially available dental impression. (b) Extracted FB. The two spectra coincided. The vertical and horizontal axes show the transmittance (%) and wave number (cm^−1^), respectively.

*Chemical Analysis*: The extracted specimen was sliced into elastic disks, and its infrared spectrum was obtained using the attenuated total reflection method of infrared absorption spectrometry. *Results*: The spectra of the extracted foreign material and the denture stabilizer did not match and were determined to be different. Subsequent analysis with several candidates, including organic compounds, showed a perfect match with commercially available silicone rubber (Fig. 3). Silicone rubbers are broadly classified into condensation curing and addition-reaction types, which use tin and platinum compounds as catalysts for curing, respectively. Elemental analysis was performed using X-ray fluorescence diffraction to distinguish the types. The specimen was analyzed using a ZSX Primus II wavelength dispersive X-ray fluorescence analyzer (measurement elements: Platinum (Pt), Stannum (Sn); X-ray tube: rhodium; measurement diameter: 20 mm). *Results*: Only platinum was detected, indicating that the FB was made of polyaddition platinum silicone instead of condensation curing silicone (Fig. 4). Since caulking agents contain Pt and Sn, this possibility was ruled out. We then conducted infrared absorption spectroscopy on another several candidates, and the resulting spectra matched the dental impression made of polyaddition silicone rubber perfectly (Fig. 5).

## Discussion

The types of aspirated/ingested FBs vary by age group. The most common FBs ingested by infants and children, and adults are legumes and fish and chicken bones, respectively [1]. In the elderly, the ingestion of dental-related items, such as dentures, and medication-related items, such as press-through packs (PTPs), increases [1]. In the very elderly, unexpected FBs in addition to dentures and PTPs are accidentally swallowed when accompanied by cognitive decline [2].

The distance an errant FB travels depends on its shape. For example, a fixed denture with a hooked denture or a sharp PTP can lodge in the esophagus or stomach, and most are removed endoscopically [2]. By contrast, the endoscopic removal of FBs crossing the pylorus is difficult; therefore, FBs are closely monitored as they travel through the anus, regardless of their shape. Thus, most FBs are excreted spontaneously [1], but small, sharp FBs may cause peritonitis due to intestinal tract perforation or abscess formation due to perforation [3, 4]. FBs made of soft, viscous, and sticky materials can cause intestinal obstruction [5]. Velitchkov [6] reviewed 14 cases of small intestinal FBs and reported that the decision for surgery should not be delayed if the FB did not migrate after 24–48 h of follow-up. Recently, the number of attempts to remove FBs using a double-balloon small bowel endoscope has been increasing, and most cases of successful removal involve a capsule endoscope left behind and not a FB mistakenly swallowed [7].

We performed infrared absorption spectrum analysis on several candidate materials and matched them with the removed FB, and a perfect match was found with the silicone rubber waveform. Silicone rubber is a low-viscosity liquid before curing and can be roughly classified into two types: condensation and addition types, which use tin and platinum as curing catalysts, respectively [8]. First, we assumed that the foreign substance was a tin-containing condensation-type caulking agent used to repair water leaks in bathrooms, etc., because of its irregular shape. Therefore, we performed an X-ray fluorescence diffraction analysis to determine whether the foreign material contained tin or platinum. And we found that the FB was only detected platinum.

The foreign substance was a denture impression, indicating that accidental ingestion occurred during denture production. The most common materials ingested accidentally in dentistry are dental fillings and crowns [9, 10]. Because of their size, many are excreted spontaneously. There has been one reported case of intestinal obstruction due to accidentally swallowing the mold of the patient’s teeth during a dental procedure [11]. The FB was too large to be a piece of a denture impression. Regarding large dental impressions, the dentist would naturally have taken precautions; however, patients with cognitive decline might swallow anything without the dentist noticing.

The time it takes for symptoms to appear after ingestion of a FB varies [12–14]. In the present, more than one year had passed since the denture, and initially it was thought that this was too long for denture impression to remain in the intestinal tract. However, there is a report that a denture was removed [15] from the small intestine after 6 years of asymptomatic life following the accidental ingestion.

This case highlights the importance of considering unusual foreign bodies, particularly among the elderly with cognitive decline, and underscores the need for awareness in both medical and dental practices to prevent such incidents.
